# Increased serum levels of anti-ganglioside M1 auto-antibodies in autistic children: relation to the disease severity

**DOI:** 10.1186/1742-2094-8-39

**Published:** 2011-04-25

**Authors:** Gehan A Mostafa, Laila Y AL-ayadhi

**Affiliations:** 1 Autism Research and Treatment Center, AL-Amodi Autism Research Chair, Department of Physiology, Faculty of Medicine, King Saud University, Riyadh, Saudi Arabia; 2 Department of Pediatrics, Faculty of Medicine, Ain Shams University, Cairo, Egypt

**Keywords:** anti-ganglioside antibodies, autism, autoimmunity, Childhood Autism Rating Scale

## Abstract

**Background:**

Autoimmunity to the central nervous system (CNS) may play a pathogenic role in a subgroup of patients with autism. This study aimed to investigate the frequency of serum anti-ganglioside M1 auto-antibodies, as indicators of the presence of autoimmunity to CNS, in a group of autistic children. We are the first to measure the relationship between these antibodies and the degree of the severity of autism.

**Methods:**

Serum anti-ganglioside M1 antibodies were measured, by ELISA, in 54 autistic children, aged between 4 and 12 years, in comparison to 54 healthy-matched children. Autistic severity was assessed by using the Childhood Autism Rating Scale (CARS).

**Results:**

Autistic children had significantly higher serum levels of anti-ganglioside M1 antibodies than healthy children (P < 0.001). The seropositivity of anti-ganglioside M1 antibodies was found in 74% (40/54) of autistic children. Serum levels of anti-ganglioside M1 antibodies were significantly higher in autistic children with severe autism (63%) than those with mild to moderate autism (37%), P = 0.001. Moreover, serum anti-ganglioside M1 antibodies had significant positive correlations with CARS (P < 0.001).

**Conclusions:**

Serum levels of anti-ganglioside M1 antibodies were increased in many autistic children. Also, their levels had significant positive correlations with the degree of the severity of autism. Thus, autism may be, in part, one of the pediatric autoimmune neuropsychiatric disorders. Further wide-scale studies are warranted to shed light on the possible etiopathogenic role of anti-ganglioside M1 auto-antibodies in autism. The role of immunotherapy in autistic patients who have increased serum levels of anti-ganglioside M1 antibodies should also be studied.

## Background

Autism is a severe neurodevelopmental disorder that is characterized by impairment in verbal and non-verbal communication, imagination and reciprocal social interaction. The prevalence of autism has surged in recent years. The etiology of autism is not well understood. Autism may occur as a result of exposure to environmental factors in the presence of genetic predisposition [1].

A possible role of immune system abnormalities in the pathogenesis of some neurologic disorders, including autism, was recently postulated. Autoimmunity to the central nervous system (CNS) is the commonest of these abnormalities [2,3]. The most important clue for the possible role of autoimmunity in autism is the presence of brain-specific auto-antibodies in many autistic children [3,4]. Other clues for the occurrence of autoimmunity in autism include; the increase of autoimmune disorders among autistic families [5-9]. Also, there is a strong association between autism and the major histocompatibility complex for the null allele of C4B in class III region. This results in low production of C4B protein leading to repeated infections which play an important role in the development of autoimmunity [10,11]. In addition, in some autistic children there is an imbalance of T-helper (Th)1/Th2 subsets toward Th2, which are responsible for the allergic response and the production of antibodies [2]. Furthermore, a new form of inflammatory bowel disease, known as ileocolonic lymphonodular hyperplasia or autistic enterocolitis, was reported in some autistic children leading researchers to suspect a gut-brain connection in autism [12].

Gangliosides are a family of sialylated glycosphingolipids expressed in the outer leaflet of the plasma membrane of the cells of all vertebrates. They are particularly abundant in the nervous system and they are involved in neurotransmission [13-16]. Gangliosides are thought to play important roles in memory formation, neuritogenesis, synaptic transmission, and other neural functions [17,18]. The administration of exogenous gangliosides seems to improve nerve regeneration [19]. Ganglioside M1 is the most abundant ganglioside in neural membranes. It may be an autoantigen through the galactose-galactosamine part of its sugar moiety [20]. In humans, gangliosides elicit a T-cell independent IgM response [21].

In immune-mediated neurological disorders, various antibodies against neuronal tissues have been discovered. Some of these antibodies have been found to correlate with the pathomechanism of the disease [22]. The key to establish the immunopathogenic role of the brain auto-antibodies is to determine their effects on specific brain functions[23].

Circulating anti-ganglioside M1 auto-antibodies may play an etiopathogenic role in some autoimmune neurological disorders such as neuropsychiatric systemic lupus erythematosus. They may play a role in the cognitive dysfunction found in some lupus patients [24]. Furthermore, in axonal Guillain-Barré syndrome, antiganglioside antibodies may be produced by the mechanism of molecular mimicry between gangliosides in the axon and lipooligosaccharides of the antecedent infectious pathogen [22,25].

Since autism may be one of the pediatric autoimmune neuropsychiatric disorders, this study aimed to investigate the frequency of serum anti-ganglioside M1 auto-antibodies, as indicators of the presence of autoimmunity to brain, in a group of autistic children. In addition, we are the first to measure the relationship between these antibodies and the degree of the severity of autism which was assessed by using the Childhood Autism Rating Scale (CARS).

## Methods

### Study population

This case-control study was conducted on 54 children who had classic-onset autism, over a period of 6 months from the beginning of August 2010 to the end of January 2011. The autistic group comprised 47 male and 7 female patients recruited from the Autism Research and Treatment Center, Faculty of Medicine, King Saud University, Riyadh, Saudi Arabia. Patients fulfilled the criteria for the diagnosis of autism according to the 4th edition of the Diagnostic and Statistical Manual of Mental Disorders [26]. Their ages ranged between 4 and 11 years [median (IQR) = 9 (3) years]. Patients who had associated neurological diseases (such as cerebral palsy and tuberous sclerosis) or metabolic disorders (such as phenylketonuria) were excluded from the study.

The control group comprised 54 age- and sex-matched apparently healthy children. They were the healthy older siblings of the healthy infants who attend the Well Baby Clinic, King Khalid University Hospital, Faculty of Medicine, King Saud University, Riyadh, Saudi Arabia for routine follow up of their growth parameters. The control children were not related to the children with autism, and demonstrated no clinical findings suggestive of immunological or neuropsychiatric disorders. Their ages ranged between 4 and 11 years [median (IQR) = 9 (4) years].

This study was approved by the local Ethical Committee of the Faculty of Medicine, King Saud University, Riyadh, Saudi Arabia. In addition, an informed written consent of participation in the study was signed by the parents or the legal guardians of the all studied subjects.

### Study measurements

#### Clinical evaluation of autistic patients

This was based on clinical history taking from caregivers, clinical examination and neuropsychiatric assessment. In addition, assessment of the degree of the severity of autism was done by using the Childhood Autism Rating Scale (CARS) [27] which rates the child on a scale from one to four in each of fifteen areas (relating to people; emotional response; imitation; body use; object use; listening response; fear or nervousness; verbal communication; non-verbal communication; activity level; level and consistency of intellectual response; adaptation to change; visual response; taste, smell and touch response and general impressions). According to this scale, children who have scored 30-36 have mild to moderate autism (n = 20, 37%), while those with scores ranging between 37 and 60 points have severe autism (n = 34, 63%).

#### Measurement of serum anti-ganglioside M1 antibodies

This was done by using ELISA kit for the specific measurement of human total anti-ganglioside M1 in cell culture supernates, serum, and plasma (Uscnlife Science and Technology Co., LTD). Monoclonal antibodies specific for ganglioside M1 had been pre-coated onto a microplate. Standards and samples were pipetted into the wells and any ganglioside M1 present was bound by the immobilized antibody. An enzyme-linked polyclonal antibody specific for gaglioside M1 was added to the wells. Following a wash to remove any unbound antibody-enzyme reagent, a substrate solutionwas added to the wells Color development was in proportion to the amount of ganglioside M1 which was bound in the initial step. The intensity of the color was measured. To increase accuracy, all samples were analyzed twice in two independent experiments to assess inter-assay variations and to ensure reproducibility of the observed results (P > 0.05). No significant cross-reactivity or interference was observed.

### Statistical analysis

The results were analyzed by a commercially available software package (Statview; Abacus Concepts, Inc., Berkley, CA, USA). The data were non-parametric, thus they were presented as median and interquartile range (IQR) which are between the 25^th ^and 75^th ^percentiles. Mann-Whitney test was used for comparison between these data. Chi-square test was used for comparison between qualitative variables of the studied groups. Spearman's rank correlation coefficient "r" was used to determine the relationship between different variables. For all tests, a probability (P) of less than 0.05 was considered significant. The highest cut-off value of serum anti-ganglioside M1 antibodies was 233 ng/ml (the 95^th ^percentile of the control values as data distribution was non-parametric).

## Results

Autistic children had significantly higher serum levels of anti-ganglioside M1 antibodies [median (IQR) = 422 (486) ng/ml] than healthy controls [median (IQR) = 43 (75) ng/ml], P < 0.001 (table 1).

**Table 1:** Serum levels of anti-ganglioside M1 antibodies in autistic patients and healthy children

	Serum anti-ganglioside antibodies Median(IQR)	Z (P)
Healthy children	43 (75)	6.43
Autistic patients	422 (486)	(0.001)
Male autistic patients	356 (355)	4.22
Female autistic patients	875 (118)	(< 0.001)

IQR, interquartile range.

According to the highest cut-off value of serum anti-ganglioside M1 antibodies, Increased serum levels of anti-ganglioside M1 antibodies were found in 74% (40/54) of all autistic children, in 55% (11/20) of patients with mild to moderate autism and in 85% (29/34) of children with severe autism.

**Figure 1:**
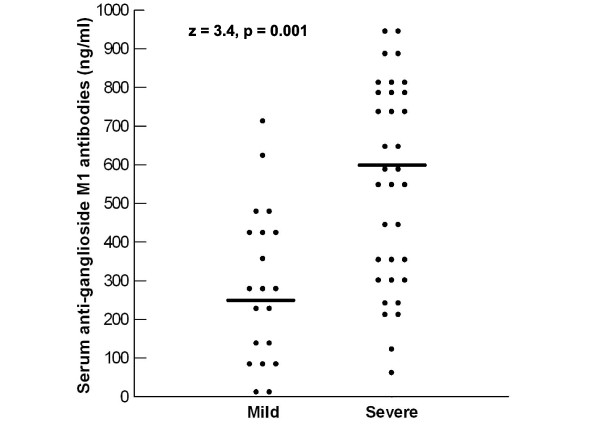
**Serum levels of anti-ganglioside M1 antibodies in relation to the degree of the severity of autism**. Median values are represented as horizontal bars.

**Figure 2:**
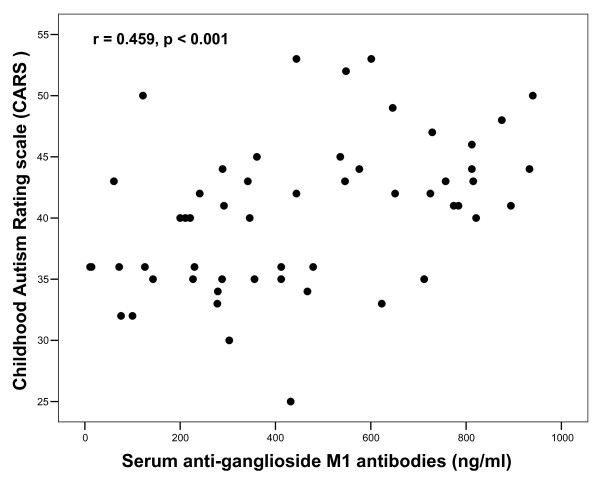
**Positive correlations between serum levels of anti-ganglioside M1 antibodies and the degree of the severity of autism**. CARS, the Childhood Autism Rating Scale.

Children with severe autism had significantly higher serum levels of anti-ganglioside M1 antibodies [median (IQR) = 592 (491) ng/ml] than patients with mild to moderate autism [median (IQR) = 278.5 (321) ng/ml], P = 0.001 (figure 1). In addition, the frequency of seropositivity of anti-ganglioside M1 antibodies was significantly higher in patients with severe autism (85%) than children with mild to moderate autism (55%), P = 0.01 (table 2). Moreover, serum levels of anti-ganglioside M1 antibodies of autistic patients had significant positive correlations with CARS, P < 0.001 (figure 2).

**Table 2:** The relationship between the frequency of the seropositivity of anti-ganglioside M1 antibodies and the degree of the severity of autism and gender of autistic patients

	Anti-ganglioside positive (n = 40)	Anti-ganglioside negative (n = 14)	**X**^**2**^ (P)
Mild to moderate autism (n = 20)	11 (55%)	9 (45%)	6.02
Severe autism (n = 34)	29 (85.3%)	5 (14.7%)	(0.01)
Male autistic patients (n = 47)	33 (70%)	14 (30%)	2.82
Female autistic patients (n = 7)	7	0	(0.1)

All female autistic children had severe autism, while only 57% of male autistic patients had severe autism. Female autistic children had significantly higher serum levels of anti-ganglioside M1 antibodies than male autistic patients [median (IQR) = 875 (118) ng/ml and 356 (355) ng/ml, respectively], P < 0.001, (table 1). Although the frequency of seropositivity of anti-ganglioside M1 antibodies was higher in female autistic patients (100%) than male autistic children (70%), this difference was not statistically significant (table 2).

Serum levels of anti-ganglioside M1 antibodies had no significant correlations with the age of the autistic children (P = 0.13).

## Discussion

Aetiology of autism presents many challenging issues and it has become an area of a significant controversy. Autoimmunity may have a role in the pathogenesis of autism [2,3]. Immune system dysfunction may represent novel targets for treatment of autism [28].

In our series, autistic children had significantly higher serum levels of anti-ganglioside M1 antibodies than healthy controls (P < 0.001). In addition, The seropositivity of anti-ganglioside M1 antibodies was found in 74% of autistic children. We could not trace data in the literature concerning the frequency of serum anti-ganglioside M1 antibodies in autistic children to compare our results.

The presence of other brain auto-antibodies such as anti-myelin basic protein antibodies and anti-myelin-associated glycoprotein antibodies have been observed in some autistic children [3,4]. In addition, the extracellular mitochondrial DNA could act as an autoimmune trigger because anti-mitochondrial antibodies type 2 were recently reported in blood of some autistic children [29]. Despite of the fact that the origin of autoimmunity in autism is unknown, immune related genes on major histocompatibility complex, which have been associated with some autoimmune diseases, may play a central role in the development of autoimmunity in autism (e.g., HLA-DRB1 and C4B null alleles) [7,10,11].

Ganglioside M1 is the most abundant ganglioside in the nervous system, in particular at synapses. It is involved in the neurotransmission at the neuromuscular junction [13-16]. Indeed, due to their location in the nervous system, gangliosides could be a target molecule in the complex autoimmune response [20]. They may play a pathogenic role in autoimmune neurological disorders [30]. Anti-ganglioside M1 antibodies are commonly found in the sera of patients with autoimmune neurological disorders such as axonal Guillain-Barré syndrome [22,25] and neuropsychiatric systemic lupus erythematosus [24].

The reason behind the formation of some brain auto-antibodies in some patients with autism is not fully understood. It is speculated that an autoimmune reaction to neurons might be trigged by some cross-reacting antigens in the environment resulting in the release of neuronal antigens. These neuronal antigens may result in induction of autoimmune reactions through the activation of the inflammatory cells in genetically susceptible individuals. The environmental antigens may include food allergies to certain peptides such as gliadin, cow's milk protein and soy [31] infectious agents [5], heavy metals such as mercury [32] and Heavea Brasiliensis proteins in natural rubber latex [33]. Cross-reacting antigens in the environment may increase some adhesion molecules on the brain endothelial cells. Pre-existing autoreactive T-cells may transmigrate across the blood-brain barrier (BBB) and induce activation of local antigen-presenting cells with production of cytokines that may result in oligodendrocyte damage and demyelination. These events may result in the release of antigens from neurofilaments that enter the circulation and induce the formation of plasma cells which produce antibodies against neuron-specific antigens. These antibodies may cross the BBB and combine with brain tissue antigens forming immune complexes that further damage the neurological tissue [34].

In the present work, children with severe autism had significantly higher serum levels of anti-ganglioside M1 antibodies than patients with mild to moderate autism, P = 0.001. In addition, the frequency of seropositivity of anti-ganglioside M1 antibodies was significantly higher in patients with severe autism (85%) than children with mild to moderate autism (55%), P = 0.01. Moreover, serum levels of anti-ganglioside M1 antibodies of autistic patients had significant positive correlations with CARS, P < 0.001. This may indicate that the extent of the elevation of serum anti-gnglioside M1 antibodies was possibly linked to the degree of the severity of autism assessed by CARS. All female patients had severe autism, while only 57% of the male patients had severe autism, this may explain the significant increase of serum anti-ganglioside M1 antibodies in female patients compared to male patients (P < 0.001). The relationship between anti-ganglioside M1 antibodies and the severity of autism is possibly a causal one, in which these auto-antibodies might be playing a role in the pathogenesis of brain damage, the extent of which may determine the clinical severity of autism. Thus, further studies, on a large scale, are warranted to reveal if anti-ganglioside M1 antibodies have a role in the pathogenesis of autism.

Previous research reported an increased frequency of autoimmune diseases in families of autistic children compared to those of healthy control subjects [5-9]. This may be an outstanding feature among autistic patients that points to their autoimmune background; the target in their case being the developing brain. This implies that in some families, immune dysfunction, perhaps induced by certain environmental triggers, could express itself in the form of autism in one of its offsprings. The high rate of autoimmune disease in the mothers of the children with autism [5-9] could also suggest that an autoimmune process exists in the mothers that is targeted toward the developing fetus in utero. Although this would be more consistent with the female preponderance in autoimmune disorders, it does not explain the high male to-female ratio observed in autism [35].

To date, a definitive relationship between autism and autoimmunity has not been fully established. On the basis of the preliminary results reported in this study, there seems to be a suggestive evidence in support of autoimmune contributions to the pathophysiology of autism in some cases. Additional investigations designed to expand on these data are warranted.

Therapy in patients who are seropositive for serum auto-antibodies is directed at reducing the concentration of these antibodies, blocking the effector mechanisms and depleting the monoclonal B cells. The recent availability of a monoclonal antibody suppressing B-cell clones, which is not myelosuppressive and does not cause secondary malignancies, allows for early targeted intervention [36]. Preliminary results suggest that this new line of therapy is well tolerated and is promising in the treatment of some patients [37,38]. Therefore, the role of this new line of therapy in autistic patients who have increased serum anti-ganglioside M1 antibodies should be studied. The administration of exogenous gangliosides seems to improve nerve regeneration [19]. Therefore, studies concerning the possible role of the administration of exogenous gangliosides in the amelioration of some autistic manifestations should also be conducted.

## Conclusions

Serum levels of anti-ganglioside M1 antibodies were increased in many autistic children. Also, their levels had significant positive correlations with the degree of the severity of autism. Thus, autism may be, in part, one of the pediatric autoimmune neuropsychiatric disorders. Further wide-scale studies are warranted to shed light on the possible etiopathogenic role of anti-ganglioside M1 auto-antibodies in autism. The role of immunotherapy in autistic patients who have increased serum levels of anti-ganglioside M1 antibodies should also be studied.

## Abbreviations

(BBB): blood brain barrier; (CARS): Childhood Autism Rating Scale; (CNS): central nervous system; (IQR): interquartile range; (ROC): Receiver operating characteristic curve; (Th): T helper cells.

## Competing interests

The authors declare that they have no competing interests.

## Authors' contributions

Both authors designed, performed and wrote the research. In addition, both authors have read and approved the final manuscript.
